# Anti-Migratory Effect of Dipotassium Glycyrrhizinate on Glioblastoma Cell Lines: Microarray Data for the Identification of Key MicroRNA Signatures

**DOI:** 10.3389/fonc.2022.819599

**Published:** 2022-08-03

**Authors:** Gabriel Alves Bonafé, Jéssica Silva dos Santos, Anna Maria Alves de Piloto Fernandes, Jussara Vaz Ziegler, Fernando Augusto Lima Marson, Thalita Rocha, Patricia de Oliveira Carvalho, Manoela Marques Ortega

**Affiliations:** ^1^ Laboratory of Cell and Molecular Tumor Biology and Bioactive Compounds, Post Graduate Program in Health Science, São Francisco University, Bragança Paulista, São Paulo, Brazil; ^2^ Laboratory of Human and Medical Genetics, Post Graduate Program in Health Science, São Francisco University, Bragança Paulista, São Paulo, Brazil; ^3^ Multidisciplinary Research Laboratory, Post Graduate Program in Health Science, São Francisco University, Bragança Paulista, São Paulo, Brazil; ^4^ Verdi Cosmetics Limited Liability Company, Joanópolis, São Paulo, Brazil; ^5^ Post Graduate Program in Biomaterials and Regenerative Medicine, Faculty of Medical Sciences and Health, Pontifical Catholic University of São Paulo, Sorocaba, São Paulo, Brazil

**Keywords:** glioblastoma, dipotassium glycyrrhizinate, nuclear factor kappa B pathway, microRNA signature, miR-4443 and miR-3620, metastasis formation inhibition

## Abstract

The nuclear factor kappa B (NF-κB) pathway has been reported to be responsible for the aggressive disease phenomenon observed in glioblastoma (GBM). Dipotassium glycyrrhizinate (DPG), a dipotassium salt of glycyrrhizic acid isolated from licorice, has recently demonstrated an anti-tumoral effect on GBM cell lines U87MG and T98G through NF-κB suppression by *IRAK2*- and *TRAF6*-mediating microRNA (miR)-16 and miR-146a, respectively. Thus, the present study aimed to evaluate the expression profiles of miRNAs related to NF-κB suppression in T98G GBM cell line after DPG exposure using miRNA microarray (Affymetrix Human miRNA 4.0A), considering only predicted miRNAs as NF-κB regulator genes. Additional assays using U251 and U138MG cells were performed to validate the array results. DPG cytotoxicity was determined by (4,5-dimethylthiazol-2-yl)-2,5-diphenyl tetrazolium bromide assay, and cellular apoptosis was quantified by DNA fragmentation and terminal deoxynucleotidyl transferase dUTP nick-end labeling (TUNEL) assay. The anti-proliferative effect was observed by cell proliferation and wound-healing assays, and the sphere formation assay examined whether DPG reduced stem cell subpopulation formation. The most over-expressed miRNAs were miR-4443 and miR-3620. The cytotoxic effect of DPG in U251 and U138MG was observed with an IC50 of 32 and 20 mM for 48 h, respectively. The IC50 of each cell line was used in all further assays. DPG treatment-induced apoptosis is observed by DNA fragmentation and increased TUNEL-positive cells. Cell proliferation and wound-healing assays showed an anti-proliferative and anti-migratory effect by DPG on the evaluated cell lines. In addition, DPG treatment led to a 100% reduction in sphere formation. The qPCR results in U251 and U138MG cells showed that DPG increased miR-4443 (2.44 *vs*. 1.11, *p*-value = 0.11; 8.27 *vs*. 1.25, *p*-value = 0.04) and miR-3620 expression (1.66 vs. 1.00, *p*-value = 0.03; 8.47 *vs*. 1.01, *p*-value = 0.03) and decreased *CD209* (0.44 *vs*. 1.10, *p*-value = 0.03; 0.49 *vs*. 1.07, *p*-value = 0.04) and *TNC* (0.20 *vs*. 1.03, *p*-value = 0.001; 0.39 *vs*. 1.06, *p*-value = 0.01) mRNA levels compared to controls. Our results suggest that DPG inhibits cell viability by activating apoptosis and inhibiting cell proliferation and stem cell subpopulation formation through miR-4443 and miR-3620 upregulation. Both miRNAs are responsible for the post-transcriptional inhibition of NF-κB by *CD209* and *TNC* modulation.

## Introduction

Glioblastoma (GBM) cells have a high capacity to invade and infiltrate normal surrounding brain tissue aggressively, hindering complete surgical resection (1). In addition, GBM cell tumors are very resistant to radiation and cytotoxic chemotherapy (2). GBM molecular subtyping, over the past two decades, has provided predictions of evolution, common disease pathways, and better treatment options (3–8). In addition, single-cell RNA sequencing revealed that multiple subtypes could exist within a single tumor, underscoring the substantial inter- and intra-tumor heterogeneity of GBM (9). MicroRNAs (miRNAs), a class of small non-coding RNA species, have critical functions across various biological processes associated with the pathogenesis of GBM (10). The miR expression signatures also characterize the phenotypic diversity of GBM subclasses through their ability to regulate developmental growth and differentiation (10, 11). Moreover, miRNAs have been previously proven to be promising diagnostic biomarkers in GBM (11–13).

Dipotassium glycyrrhizinate (DPG, C_42_H_60_K_2_O_16_), a dipotassium salt of glycyrrhizic acid (C_42_H_62_O_16_) isolated from licorice (*Glycyrrhiza glabra*), has recently demonstrated an anti-tumoral effect on GBM cell lines U87MG and T98G through a decrease of proliferation and an increase of apoptosis. In addition, DPG anti-tumoral effect seems to be related to nuclear factor kappa B (NF-κB) pathway suppression by *IRAK2*- and *TRAF6*-mediating miR-16 and miR-146a, respectively (14). In fact, one study supported the role of miRNAs in NF-κB regulation (15).

In the present study, the expression profile of miRNAs in T98G GBM cells, a temozolomide (TMZ)-resistant cell line, after DPG exposure was obtained using microarrays. Interestingly, the most over-expressed miRNAs were miR-4443 and miR-3620. Additional cellular studies using other GBM cell lines were performed to validate the array results. Moreover, DPG decreased the viability and sphere formation of the cultured stem cell-enriched populations of established GBM cell lines.

## Materials and Methods

### Cell Culture and Treatments

U87MG, T98G, U251, and U138MG were kindly donated by Dr. Adriana da Silva Santos Duarte, Hemocenter, University of Campinas, Campinas, São Paulo, Brazil, and were cultured at 37°C in Dulbecco’s modified Eagle’s medium (DMEM) high glucose supplemented with 10% fetal calf serum (FCS) and 1% penicillin/streptomycin (Cultilab, Campinas, São Paulo, Brazil) at 37°C in a 5% CO_2_ atmosphere. For all experiments, the cells were seeded and grown for 72 h before the experimental treatments. The cells were passaged by Trypsin 0.25% (Cultilab) until the seventh passage after thawing.

DPG (chemical abstracts service number 68797-35-3) was obtained from Verdi Cosmetics LLC (Joanópolis, São Paulo, Brazil). For the cell line treatments, DPG was diluted in DMEM to prepare a 2,000-μM stock solution. All treatment assays were performed in the presence of 10% FCS and 1% P/S.

### Ultraperformance Liquid Chromatography

The composition of DPG was determined by ultraperformance liquid chromatography–mass spectrometry using an Acquity UPLC system (Waters Co., Milford, MA, USA) coupled with an Acquity TQD mass spectrometer (Micromass; Waters Co.) as described in Franco et al. (16).

### Determination of Cellular Metabolic Activity (Cell Viability)

Adherent GBM cells (U251 and U138MG) were seeded in 96-well flat-bottomed tissue culture plates (0.2 × 10^6^ cells/plate). After 24 h of incubation at 37°C in a 5% CO_2_ environment, various concentrations (5, 8, 12, 15, 18, 20, 24, 28, 32, and 36 mM) of DPG were used to treat the GBM cell lines based on a previous study (14). The cells were cultured for 24, 48, and 72 h prior to (4,5-dimethylthiazol-2-yl)-2,5-diphenyl tetrazolium bromide incubation (Sigma, St. Louis, MO, United States). The cells were incubated for 4 h at 37°C in a 5% CO_2_ environment. Following incubation, formazan crystals were solubilized with 100 μL of dimethyl sulfoxide. Cell viability was determined by measuring the optical density at 550 nm using a microplate spectrometer (Thermo Fisher, Waltham, MA, USA). The cell survival rates were expressed as percentages of the value of normal cells. Untreated control cells were analyzed in all experiments, and all DPG dose treatments were performed in triplicate.

### Determination of DPG Effects on Cell Viability

U251 and U138MG cells (0.4 × 10^6^ cells/well) were cultured in 24-well tissue culture plates for 24 h at 37°C in a 5% CO_2_ environment and further exposed to 32 and 20 mM of DPG, respectively. The cells were washed in phosphate-buffered saline (PBS), and the viable cells were counted by trypan blue dye exclusion assay for 4 days. Untreated cells were used as a control, and the experiments were performed in triplicate.

### Evaluation of the Effect of DPG on Cell Death by DNA Fragmentation

U251 and U138MG cells were cultured in 6-well tissue culture plates for 24 h at 37°C in a 5% CO_2_ environment and further exposed to 32 and 20 mM of DPG for 48 and 72 h, respectively. The TMZ-resistant U251 was also exposed for 96 h. DNA was isolated using lithium chloride extraction (17). The purity of DNA was analyzed in a spectrophotometer at 260/280 nm, and the ratio was confirmed to be between 1.7 and 1.9. DNA samples were then electrophoresed on 1.5% agarose gel and visualized with ethidium bromide staining under ultraviolet illumination.

### Evaluation of DPG Effect on Cell Death by TUNEL Assay

U251 and U138MG cells were cultured in 96-well tissue culture plates for 24 h at 37°C in a 5% CO_2_ environment and further exposed to 32 and 20 mM of DPG for 72 h, respectively. Cellular apoptosis was evaluated after treatment with DPG by *in situ* terminal deoxynucleotidyl transferase dUTP nick-end labeling (TUNEL) assay using *in situ* cell death detection kit, fluorescein (Roche Applied Science, Mannheim, Germany), according to the manufacturer’s protocols. Apoptotic indexes were calculated by scoring four randomly selected fields and counting the number of apoptotic cells over the total of viable cells, representing a quota compared to untreated cells. The cells were directly analyzed under a fluorescence microscope (Axio Vert. A1 ZEISS, Germany).

### Wound Healing Assay

U251 and U138MG cells were seeded in 6-well plates (1 × 10^6^ cells/well) and grown overnight at 37°C in a 5% CO_2_ environment to confluence. The monolayer of cells was scratched with a 200-mL pipette tip to create a wound, and the plates were washed twice with PBS and cultured with DPG (32 mm and 20 mM, respectively). Afterward, cells migrating from the leading edge were photographed at 0, 24, 48, and 72 h under an inverted microscope (Axio Vert. A1 ZEISS). Untreated cells were used as a control. The distance of the scratch closure was examined using ImageJ software (National Institutes of Health, Bethesda, MD, United States). Each value is derived from the same selected fields, and results are expressed as the mean of migrating cell numbers per field.

### Sphere-Cultured Stem Cell-Enriched GBM Populations

U251 and U138MG cells (1 × 10^4^ cells/well) were cultured at 37°C in a 5% CO_2_ environment in serum-free DMEM/F12 supplemented with N_2_ supplement (StemCell, Vancouver, Canada) containing epidermal growth factor (20 ng/mL) (Peprotech, Ribeirão Preto, São Paulo, Brazil), basic fibroblast growth factor (20 ng/mL) (Peprotech), and 1% penicillin/streptomycin (120 mg/mL) (Thermo Fisher) for at least 6 days. For the subsequent DPG treatment (32 and 20 mM, respectively), 75-µm neuro-spheres were cultured for 24 and 48 h. Cells without DPG were cultured as controls. Cells were observed and photographed under an inverted microscope (Axio Vert. A1 ZEISS).

### miRNA and Gene Expression Arrays

miRNA expression profiles were conducted in T98G cell line DPG exposure (24 mM; 48 h of DPG treatment) (14) and control cells. Analyses were performed using Affymetrix^®^ GeneChip miRNA 2.0 array (Affymetrix, Santa Clara, CA, USA), which detects 2,578 known human miRNAs (miRBase v.15; Affymetrix). Total RNA was labeled with FlashTag Biotin HSR, hybridized with the arrays, then washed with PBS, stained, and scanned according to Affymetrix GeneChip Command Console software. The miRNA QC Tool software (Affymetrix) was used for data summarization, normalization, and quality control.

### Identification of miRNA Target Genes

The analysis of miRNA differential expression profile was performed considering only 91 miRNAs previously selected and predicted as regulators of genes involved in the NF-κB pathway (**Supplementary Table S1)**. Conventional online programs, including miRanda (http://www.microrna.org), TargetScan (http://www.targetscan.org), and Findtar (http://bio.sz.tsinghua.edu.cn), were used to predict the targets of miRNAs. To identify the most likely targets, mRNA and miRNA expression data obtained on the same biological samples using Microsoft Excel tools were integrated. Twofold upregulated miRNA and corresponding 2fold downregulated mRNA targets were selected for further investigation.

### Total RNA Isolation

For the U87MG and T98G cells, the half-maximal inhibitory concentration (IC50) used is based on a previous study using DPG as a therapeutic compound in GBM cell lines (14). Total RNA was isolated from U87MG, T98G, U251, and U138MG cells after DPG exposure (18, 24, 32, and 20 mM for 48 h, respectively) and control cells using TRIzol^®^ reagent (Thermo Fisher), according to the manufacturer’s instructions. Total RNA quantification was performed using the ND-1000 spectrophotometer (Nanodrop; Thermo Fisher).

### Validation of miRNA and mRNA Expression Levels

cDNA conversion was performed using the High-Capacity cDNA Reverse Transcription Kit (Applied Biosystems, Foster City, CA, USA). qPCR was performed on a 7500 Fast Real-Time PCR system (Applied Biosystems) using SYBR-Green PCR Master Mix (Applied Biosystems). Each sample was examined in triplicate, and the expression of each gene was normalized by control gene expression (*GAPDH*) and calculated by applying the 2^−ΔΔCt^ method (18). The expression value of each gene was represented as fold change. The primer sequences used for amplification by qPCR with SYBRGreen dye (Applied Biosystems) are as follows: *CD209* 5′-CATGTCTAACTCCCAGCGG-3′ (sense) and 5′-GAAA GTCCCATCCAGGTGAAG-3′ (anti-sense), *TNC* 5′-CACT ACAC AGCC AAGATCCAG-3′ (sense) and 5′-TCGT GTCT CCATT CAGC ATTG-3′ (anti-sense), and *GAPDH* (forward) 5′- CCAC TTG ATTTTGGAGGGAT-3′ and (reverse) 5′-GCA CCGT CAAG GCTGAGAAC-3′.

miRNA expression analysis was validated using the MicroRNA Assay kit (Applied Biosystems), which incorporates a target-specific stem-loop reverse transcription primer to provide specificity for mature miRNA target. U6 served as an endogenous control for the normalization of RNA input. Specific primers for mRNA expression analysis and the endogenous control were provided by Thermo Fischer (miR-4443: assay 463010_mat; miR-3620: assay CTKA3MT; and U6: assays 001973 and 03928990_g1). The specificity of the PCR products was tested by dissociation curves. Relative values of transcripts were calculated using the equation 2^−ΔΔCt^, where ΔCt is equal to the difference in threshold cycles for target and reference genes. Each experiment was performed in triplicate.

### Statistical Analysis

A two-tailed *T*-test was performed for all two sets of numerical data (treated and non-treated cells), and *P*-value ≤0.05 was considered statistically significant. The results are expressed as mean ± SD from experiments repeated at least three times. Statistical analysis was performed using the Statistical Package for the Social Sciences software (IBM SPSS Statistics for Macintosh, version 27.0.).

## Results

### DPG Analysis by Liquid Chromatography

Mass spectrometry (UPLC-QTOF, Waters) was applied to evaluate the presence of DPG in the sample used for the present study. The signals from [M-H]^-^ and [M-2H]^-2^ anions as well as the mass measurement accuracy and the adequacy of the simulated isotopic standard for [M-H]^-^ ion confirmed the presence of the DPG (**Supplementary Figure S1A**).

### DPG Inhibits Cell Viability and Proliferation

**Figure 1:**
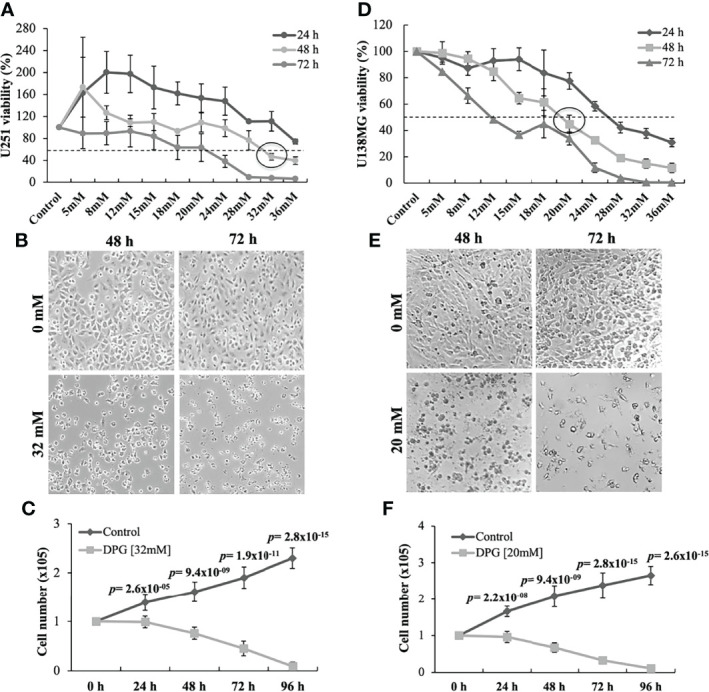
Dipotassium glycyrrhizinate (DPG) reduces cell viability and changes morphology in glioblastoma cell lines. **(A)** U251 cells were treated with different concentrations of DPG for 24, 48, and 72 h and evaluated by the 4,5-dimethylthiazol-2-yl)-2,5-diphenyl tetrazolium bromide (MTT) assay to determine the IC50 (32 mM for 48 h). **(B)** Morphological and nuclear changes were observed in U251 cells, especially after 72 h of exposure to DPG, when compared to untreated cells. **(C)** The DPG treatment also significantly inhibited the rate of proliferation in U251 cells in a time-dependent way compared to untreated cells. **(D)** U251 cells were treated with different concentrations of DPG for 24, 48, and 72 h and evaluated by the MTT assay to determine the IC50 (20 mM for 48 h). **(E)** Morphological and nuclear changes were observed in U138MG cells, especially after 72 h of exposure to DPG, when compared to untreated cells. **(F)** The DPG treatment significantly inhibited the rate of proliferation in U138MG cells in a time-dependent way compared to untreated cells. The data presented are the mean ± standard deviation of the experiments performed in triplicate. The *T*-test indicated *p*-values ≤0.05.

The antitumor effect of DPG was evaluated by using two GBM cell lines (U251 and U138MG). Based on these findings, the cytotoxic effect of DPG was time and dose dependent, and the IC50 in U251 and U138MG was 32 and 20 mM for 48 h, respectively (**Figures 1A, D**). For all further assays, IC50 was adopted. Moreover, it was observed that cells presented nuclear morphological changes after 48 h of DPG treatment (**Figures 1B, E**). Furthermore, cell proliferation assay showed a significantly anti-proliferative effect by DPG on the same cell lines starting after 24 h of treatment (*p*-value = 2.6 × 10^-5^ and *p*-value = 2.2 × 10^-8^, respectively) until 96 h later (*p*-value = 2.8 × 10^-15^ and *p*-value = 2.6 × 10^-15^, respectively) (**Figures 1C, F**).

### DPG Induces Apoptosis

**Figure 2:**
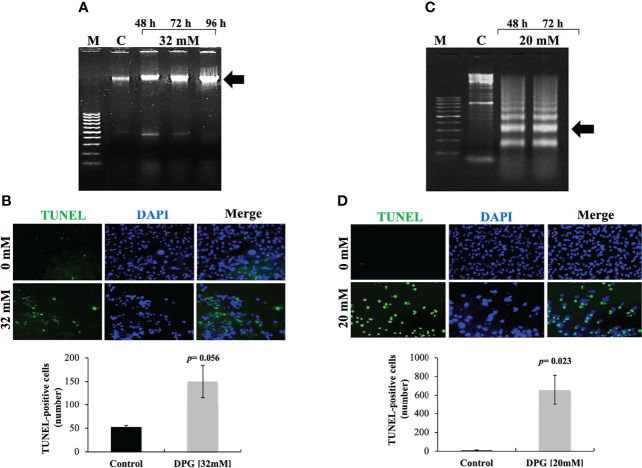
Apoptotic effects of dipotassium glycyrrhizinate (DPG) on glioblastoma cell lines. **(A)** U251 cells were incubated with 32 mM of DPG for 48, 72, and 96 h. Genomic DNA was isolated and analyzed on 1.5% agarose gel stained with ethidium bromide. M, DNA marker 100 base pairs; C, untreated control cells. **(B)** U251 cells were treated with DPG (32 mM for 96 h). After incubation with DPG, TUNEL-positive cells were quantified by the ImageJ computer program. **(C)** U138MG cells were incubated with 20 mM of DPG for 48 and 72 h. Genomic DNA was isolated and analyzed on 1.5% agarose gel stained with ethidium bromide. M, DNA marker 100 base pairs; C, untreated control cells. **(D)** U138MG cell lines were treated with DPG (20 mM for 72 h). After incubation with DPG, TUNEL-positive cells were quantified by the ImageJ computer program. The data presented are mean ± standard deviation of the experiments performed in triplicate. The results showed that treatment with DPG increased the number of cells undergoing apoptosis compared to untreated control cells (U251: *p*-value = 0.056; U138MG: *p*-value = 0.023).

To investigate cellular apoptosis, DNA fragmentation was quantified by TUNEL assay. As expected, DPG was also able to induce cellular apoptosis by DNA fragmentation (**Figures 2A, C**), which was confirmed by increased TUNEL-positive cells when compared to untreated cells (**Figures 2B, D**), mostly on the U138MG cell line (*p*-value = 0.023).

### DPG Inhibits Invasion and Migration

**Figure 3:**
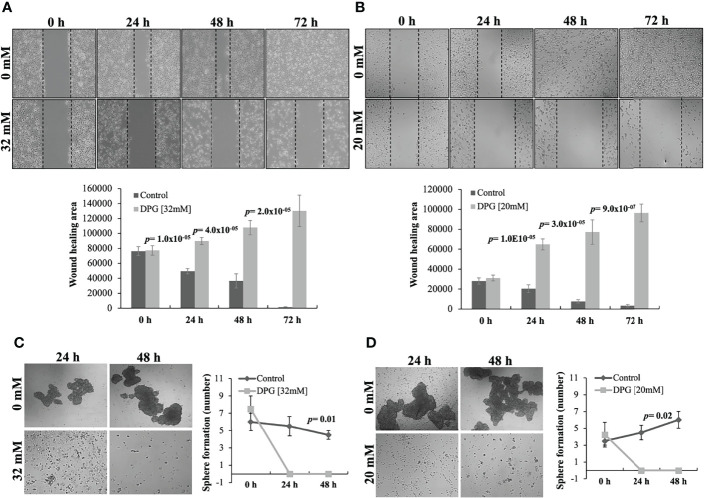
Dipotassium glycyrrhizinate (DPG) inhibits cell migration and cancer stem-like cells in glioblastoma cell lines. **(A)** U251 and **(B)** U138MG cell lines treated with DPG fill the wound area (the area between the two dotted lines) more slowly at 24, 48, and 72 h when compared to untreated control cells (*p*-value ≤ 0.001). The wound-healing assay was quantified using the ImageJ computer program by measuring the relative area of the wound after treatment with DPG. Loss of sphere-forming capacity of cell lines **(C)** U251 (*p*-value = 0.01) and **(D)** U138MG (*p*-value = 0.02) after 24 and 48 h of treatment with DPG when compared to untreated control cells. The data presented are the mean ± standard deviation of the experiments performed in triplicate.

Furthermore, to investigate the effect of DPG on the migration ability of U251 and U138MG, both cell lines were treated with DPG for 24, 48, and 72 h, and a wound-healing motility assay was performed simultaneously. The results showed that cells exposed to DPG migrated significantly slower than DPG-free control cells starting after 24 h of treatment (*p*-value = 1 × 10^-5^ and *p*-value = 1 × 10^-5^, respectively) until 96 h later (*p*-value = 2 × 10^-5^ and *p*-value = 9 × 10^-7^, respectively) (**Figures 3A, B**).

### DPG Effect on GBM Stem-Like Cells

The phenotypic plasticity of cancer cells grown as neurospheres to elucidate the influence of DPG on GBM stem-like cells was also investigated. Thus, it was observed that DPG promoted a 100% reduction in neurosphere formation compared to untreated cells, starting after 24 h of DPG exposure (*p*-value ≤ 0.05) (**Figures 3C, D**), characterizing DPG as an antitumor compound.

### Identification of miRNAs With Differential Expression

Global miRNA expression changes in T98G cells were evaluated after 48 h of treatment with 24 mM of DPG. No treated T98G cells were evaluated as control. A total of 11 miRNAs with DE-miRNAs were identified after comparing DPG-treated and control samples (among 91 predicted miRNAs as NF-κB regulator genes) (**Supplementary Figure S2A**). The number of upregulated and downregulated DE-miRNAs is shown in **Tables 1**, **2**, respectively. Furthermore, a new analysis was performed considering the expression values, mRNA volume, and target genes predicted to be involved in GBM pathogenesis, and most DE-miRNAs were found to be miR-4443 and miR-3620 (**Supplementary Figure S2B**) and their respective target genes *CD209* and *TNC*.

**Table 1:** The most upregulated microRNAs (FC ≥-2) after dipotassium glycyrrhizinate (DPG) treatment in the glioblastoma T98G cell line by global miRNA microarrays.

	MicroRNAs	FC	mRNA volume	Target genes (>99%)^a^	Function
1	miR-4448	16.70	2.01	*CASP4*	Apoptosis—caspase
2	miR-1587	3.88	3.60	*TNC*	Cell adhesion—control of cell growth, migration, and adhesion by the ECM protein
3	miR-3620-5p	2.04	6.68	*TNC*	Cell adhesion—control of cell growth, migration, and adhesion by the ECM protein
4	miR-4443	2.10	7.12	*CD209*	Cell adhesion—dendritic cell surface by leptin C
5	miR-7111-5p	2.66	2.71	*MAP4K1*	Stress response—activator of the stress-induced protein kinase pathway
6	miR-3148	6.79	2.93	*S100A4*	Miscellaneous—tumor suppressor

FC, fold change; mRNA volume, $\;\sqrt{\text{normalized control value }\times \text{normalized DPG value}}$ .

a Prediction according to TargetScan database.

**Table 2:** The most downregulated microRNAs (FC ≥-2) after dipotassium glycyrrhizinate (DPG) treatment in the glioblastoma T98G cell line by global miRNA microarrays.

	MicroRNAs	FC	mRNA volume	Target genes^a^	Function
1	miR-27b-3p	-2.22	4.21	*BCL3*	Transcription—coactivator for NF-κB p50 and p52
2	miR-106a-5p	-6.13	4.97	*MMP3*	Enzymes—related to metastasis
3	miR-17-5p	-4.09	5.39	*MMP3*	Enzymes—related to metastasis
4	miR-20a-5p	-4.38	3.61	*MMP3*	Enzymes—related to metastasis
5	miR-22-3p	-2.46	5.06	*PTEN*	Miscellaneous—tumor suppressor

FC, fold change; mRNA volume, $\;\sqrt{\text{normalized control value }\times \text{normalized DPG value}}$ .

a Prediction according to TargetScan database.

### Validation of miRNA and mRNA Expression Levels

**Figure 4:**
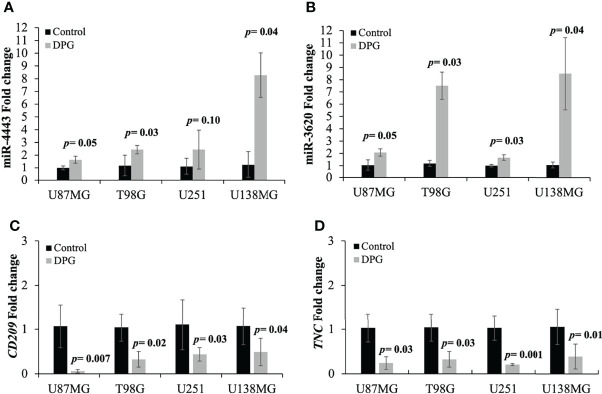
Dipotassium glycyrrhizinate (DPG) modulated the expression of selected miRNAs and their respective target genes in glioblastoma cell lines (GBM). **(A)** U87MG (1.64 *vs*. 1.00, *p*-value = 0.05), T98G (2.44 *vs*. 1.17, *p*-value = 0.03), U251 (2.44 *vs*. 1.11, *p*-value = 0.11), and U138MG 8.27 *vs*. 1.25, *p*-value = 0.04) cell lines treated with DPG showed an increased level of miR-4443 expression compared to untreated control cells. **(B)** U87MG (2.08 *vs*. 1.04, *p*-value = 0.05), T98G (7.50 *vs*. 1.19, *p*-value = 0.007), U251 (1.66 *vs*. 1.00, *p*-value = 0.03), and U138MG (8.47 *vs*. 1.00, *p*-value = 0.03) cell lines exposed to DPG showed increased miR-3620 expression levels compared to untreated control cells. **(C)** Decreased *CD209* (0.06 *vs*. 1.07, *p*-value = 0.007; 0.32 *vs*. 1.04, *p*-value = 0.002; 0.44 *vs*. 1.11, *p*-value = 0.03; 0.49 *vs*. 1.07; *p*-value = 0.04) and **(D)** *TNC* (0.24 *vs*. 1.03, *p*-value = 0.03; 0.05 *vs*. 1.15, *p*-value = 0.03; 0.20 *vs*. 1.03, *p*-value = 0.001; 0.39 *vs*. 1.06; *p*-value = 0.01) mRNA levels were observed in U87MG, T98G, U251, and U138MG treated with DPG compared to control cells. The data presented are mean ± standard deviation of the experiments performed in triplicate.

The expression of both miRNAs and their predicted target genes was evaluated by qPCR using U87MG, T98G, U251, and U138MG cell lines after exposure to DPG. Untreated cells were used as controls. In accordance with the global analysis, the mean mRNA expression level was significantly higher in DPG-treated T98G cells when compared to control cells for miR-4443 (2.44 *vs*. 1.17, *p*-value = 0.03) (**Figure 4A**) and miR-3620 (7.50 *vs*. 1.19, *p*-value = 0.007) (**Figure 4B**). In addition, qPCR showed that DPG increased the level of miR-4443 (2.44 *vs*. 1.11, *p*-value = 0.11; 8.27 *vs*. 1.25, *p*-value = 0.04; 1.64 *vs*. 1.00, *p*-value = 0.05) (**Figure 4A**) and miR-3620 expression (1.66 *vs*. 1.00, *p*-value = 0.03; 8.47 *vs*. 1.00, *p*-value = 0.03; 2.08 *vs*. 1.04, *p*-value = 0.05) in U251, U138MG, and U87MG cell lines (**Figure 4B**).

On the other hand, the *CD209* and *TNC* expression levels were lower in DPG-treated cells when compared to controls (**Figures 4C, D** **)**. Thus, decreased *CD209* (0.06 *vs*. 1.07, *p*-value = 0.007; 0.32 *vs*. 1.04, *p*-value = 0.002; 0.44 *vs*. 1.11, *p*-value = 0.03; 0.49 *vs*. 1.07; *p*-value = 0.04) (**Figure 4C**) and *TNC* (0.24 *vs*. 1.03, *p*-value = 0.03; 0.05 *vs*. 1.15, *p*-value = 0.03; 0.20 *vs*. 1.03, *p*-value = 0.001; 0.39 *vs*. 1.06; *p*-value = 0.01) (**Figure 4D**) mRNA levels were observed in U87MG, T98G, U251, and U138MG when compared to control cells.

## Discussion

In the present study, we demonstrated first that DPG, under *in vitro* conditions, was able to significantly reduce the number of viable cells in GBM cell lines U251 and U138MG, inhibiting cell growth and adhesion. This finding corroborates with previous results using the GBM U87MG and T98G cell lines (14). In addition, this previous study has suggested alterations in miRNA expression after DPG treatment in GBM cell lines U87MG and T98G (14), which were able to modulate NF-κB genes. Thus, here a global analysis evaluated potentially over-expressed miRNAs responsible for the NF-κB pathway in GBM cells. The choice of T98G for array analysis was based on hypermethylated *MGMT* promoter and mutated *P53* gene. In fact, cell lines with a hypermethylated *MGMT* promoter and mutated *P53* gene appeared more resistant to the action of TMZ than the ones with wild-type *P53* (19) since p53 protein is fundamental in regulating the cell cycle arrest and the entry in the apoptotic process (20, 21). Besides this, it was observed previously that the effect of DPG on T98G decreased cell proliferation and increased migration and invasion at a concentration of 24 mM (14).

Few studies have focused on miRNAs involved in the NF-κB pathway, which is known as continuously active (22) in GBM (14, 23–28). Thus, the large-scale global approach evaluated 91 miRNAs previously selected and predicted as regulators of genes involved in the NF-κB pathway in T98G lineage exposed to DPG action. Among the 11 DE-miRNAs reported in the present study, none has been previously evaluated in GBM cells. Besides, 2 miRNAs (miR-4443 and mir-3620-5p) were further validated using 4 GBM cell lines. Both miRNAs are predicted to be responsible for *TNC* and *CD209* modulation, respectively. Those genes are known as post-transcriptional target genes for the NF-κB pathway.

Thus, our study revealed, for the first time, that both miR-4443 and mir-3620-5p are upregulated on GBM cell lines after DPG exposure. miR-4443 was first identified by Xun et al. (29) in enterovirus-71-infected cells. In addition, it has been shown that miR-4443 plays a role in acquiring drug resistance in breast cancer (30) and inhibits cell proliferation and metastasis in colon cancer (31). In addition, miR-4443 is decreased in metastatic and serous samples from ovarian cancer (32). In the study of Gao et al. (33), the authors have found that long non-coding RNAs (lncRNAs) MNX1-AS1 were upregulated in GBM tissues and cell lines. The knockdown of MNX1-AS1 significantly inhibited the proliferation, migration, and invasion of GBM cells. In addition, the overexpression of miR-4443 significantly inhibited the expression of MNX1-AS1 and *vice versa*. Moreover, there was an inverse correlation between the expression levels of MNX1-AS1 and miR-4443 in GBM tissues. The overexpression of miR-4443 also inhibited the proliferation, migration, and invasion of GBM cells. In contrast, the inhibition of miR-4443 reversed the effects of MNX1-AS1 knockdown on GBM cell proliferation, migration, and invasion. MNX1-AS1 promoted the proliferation, migration, and invasion of GBM cells by inhibiting miR-4443. miR-3620 was able to reverse claudin-4 upregulation in gastric cancer cell lines, which is responsible for reinforcing proliferation, invasion, and epithelial–mesenchymal transition in gastric cancer and with poor prognosis (34).

*TNC* and *CD209* genes play an important role in the progression of GBM by regulating the migration, adhesion, and invasion of tumor cells into adjacent tissues (35, 36). The *TNC* gene was evaluated in tumor tissues and primary cultures of patients with GBM, and its increased expression was associated with the process of carcinogenesis and invasion (35, 37). A recent survey of tumor tissues from patients with GBM correlated the infiltrative character of GBM malignant cells with an increased expression of *CCL15*, *CCL17*, *CD209*, and *TNF*-α genes (36). In the GBM cell line (U87MG), *CD209* gene interacts with TGF-β1 gene, stimulating cell invasion and metastasis (38). In addition, the results of the cell proliferation assay and wound healing in the present study corroborate with what was observed previously regarding *CD209* and *TNC* genes, both involved in cell metastasis process. Both genes showed a significant decrease in expression due to the overexpression of miR-4443 and miR-3620, respectively, after the DPG effect in the evaluated GBM cell lines in the study.

Therefore, DPG-modulated miRNAs are involved in the post-transcriptional inhibition of the NF-κB pathway. Interestingly, in a previous study, DPG inhibited the NF-κB pathway by modulation of miR-16 and miR-146a, which inhibited the expression of its target genes *IRAK2* and *TRAF6*, respectively (14). It appears that the antitumor action of DPG can inhibit genes that belong to the NF-κB signaling pathway and genes downstream by the overexpression of miRNAs.

In accordance with the results observed in the present study, another recent study has suggested that DPG has an apoptotic, anti-proliferative, and anti-migratory effect on the melanoma cell line (SK-MEL-28) bearing the *BRAF* mutation. DPG was also able to inhibit cancer stem-like cells that may cause cerebral tumor formation (39). In addition, DPG treatment has shown that SK-MEL-28 cells also presented a significantly higher level of miR-4443 and miR-3620 expression than control cells. In contrast, their predicted genes, *CD209* and *TNC*, significantly presented reduced mRNA levels after DPG compared to untreated cells. Furthermore, the migration of SK-MEL-28 cells stimulated by 12-O-tetradecanoylphorbol-13-acetate (TPA) was attenuated by adding DPG by wound-healing assay. In addition, the MMP-9 expression level was inhibited by DPG in melanoma cells stimulated by TPA and compared to only TPA-treated cells (39).

Therefore, in the present study, the potential inhibition by DPG was verified on miR-4443 and miR-3620, possible therapeutic targets for GBM. In addition, the results obtained in the present study may contribute to the development of future *in vivo* studies to target GBM cells by DPG, which is a promising approach to restrict tumor cell growth.

## Data Availability Statement

The raw data supporting the conclusions of this article will be made available by the authors, without undue reservation.

## Author Contributions

Conception and design: MMO; acquisition of data: GAB, JSS, and JVZ; analyses and interpretation of data: MMO and GAB; statistical analyses: FALM; drafting of the manuscript: MMO; mass spectrometer analyses: POC and AMAPF; TUNEL analysis: GAB and TR; study supervision: MMO. All authors contributed to the article and approved the submitted version.

## Funding

The fInancial support provided for this work by the São Paulo Research Foundation (FAPESP) scholarships #2018/05930-0 and grant #2015/03870-1 and by Coordination for the Improvement of Higher Education Personnel (CAPES) scholarship #88887.464813/2019-00 is gratefully acknowledged.

## Conflict of Interest

Author JZ is employed by Verdi Cosmetics LLC, Joanópolis, São Paulo, Brazil.

The remaining authors declare that the research was conducted without any commercial or financial relationships that could be construed as a potential conflict of interest.

## Publisher’s Note

All claims expressed in this article are solely those of the authors and do not necessarily represent those of their affiliated organizations, or those of the publisher, the editors and the reviewers. Any product that may be evaluated in this article, or claim that may be made by its manufacturer, is not guaranteed or endorsed by the publisher.

## Supplementary Material

Supplementary Figure 1 Mass spectrum of dipotassium glycyrrhizinate (DPG). The presence of the ion [M-H]^-^ (the anion of the DPG) and the ion [M-2H]^-2^ is highlighted. The error observed for measuring the ion [M-H]^-1^ (DPG anion) was -1.83 ppm.

Supplementary Figure 2 Identification of microRNAs (miRNAs) with differential expression (DE-miRNAs) in glioblastoma T98G cell line. **(A)** Scatter plot of 11 DE-miRNAs identified after comparing dipotassium glycyrrhizinate (DPG)-treated and control T98G cell line (among 91 predicted miRNAs as NF-κB regulator genes). The gray and red dots represent each predicted miRNA. The red dots represent those that presented |fold change, FC ≥ 2|, according to the analysis of the computer program Affymetrix GeneChip^®^ Command Console™. The dots above and below the dashed line represent those up- and downregulated miRNAs after DPG treatment, respectively. **(B)** Volume graph of the most overexpressed miRNAs among the 11 DE-miRNAs in T98G cell line exposure to DPG. Each gray, blue, and red dot represents one DE-miRNA. The red dots represent those miRNAs that presented both |FC ≥ 2| and increased volume of mRNA detected and quantified by the Affymetrix GeneChip^®^ Command Console™ software. The dots above and below the dashed line represent those up- and downregulated miRNAs after DPG treatment, respectively.

Supplementary Table 1 List of the 91 selected microRNAs predicted as NF-κB modulators and their predicted target genes (above 99%, according to TargetScan database) belonging to the NF-κB signaling pathway.

## Abbreviations

CAS, chemical abstracts service; CD209, cluster of differentiation 209; DMEM, Dulbecco’s modified Eagle’s medium; FCS, fetal calf serum; DMSO, dimethyl sulfoxide; GA, glycyrrhizic acid; DPG, dipotassium glycyrrhizinate; *IRAK2*, interleukin-1 receptor-associated kinase 2; qPCR, quantitative polymerase chain reaction; NF-κB, nuclear factor kappa B; miRNAs, microRNAs; MTT, (4,5-dimethylthiazol-2-yl)-2,5-diphenyl tetrazolium bromide; PBS, phosphate-buffered saline; SD, standard deviation; *TNC*, tenascin; TMZ, temozolomide; *TRAF6*, TNF receptor-associated factor; TUNEL, terminal deoxynucleotidyl transferase (TdT) dUTP nick-end labeling.
