# Supplementary figures and images for: Anti-Migratory Effect of Dipotassium Glycyrrhizinate on Glioblastoma Cell Lines: Microarray Data for the Identification of Key MicroRNA Signatures
Source: Front Oncol. 2022 Aug 3;12:819599. doi: 10.3389/fonc.2022.819599 (PMC9382584; doi:10.3389/fonc.2022.819599)

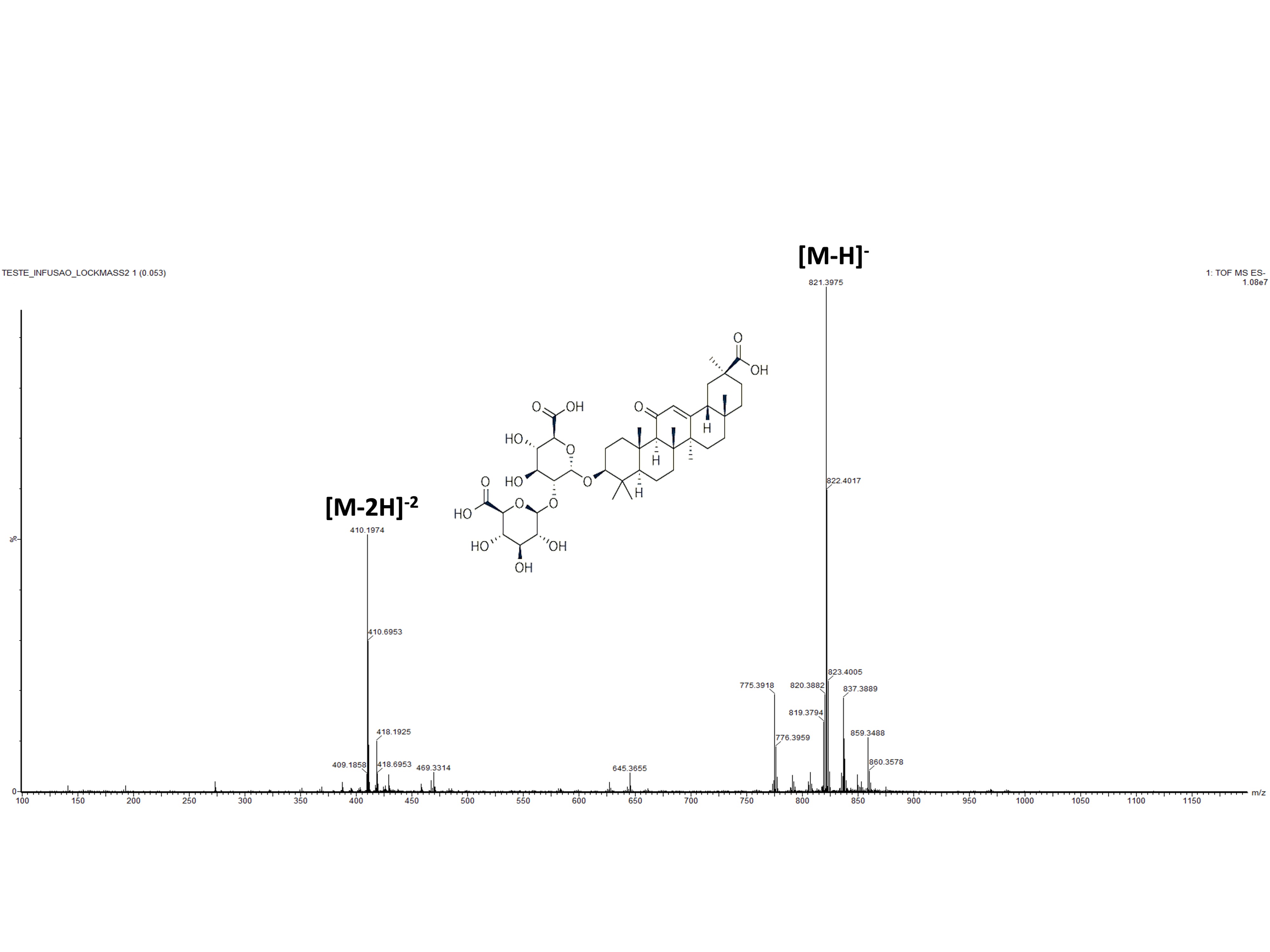

Supplement: Supplementary Figure 1 — Mass spectrum of dipotassium glycyrrhizinate (DPG). The presence of the ion [M-H]- (the anion of the DPG) and the ion [M-2H]-2 is highlighted. The error observed for measuring the ion [M-H]-1 (DPG anion) was -1.83 ppm. [file Image_1.jpeg]

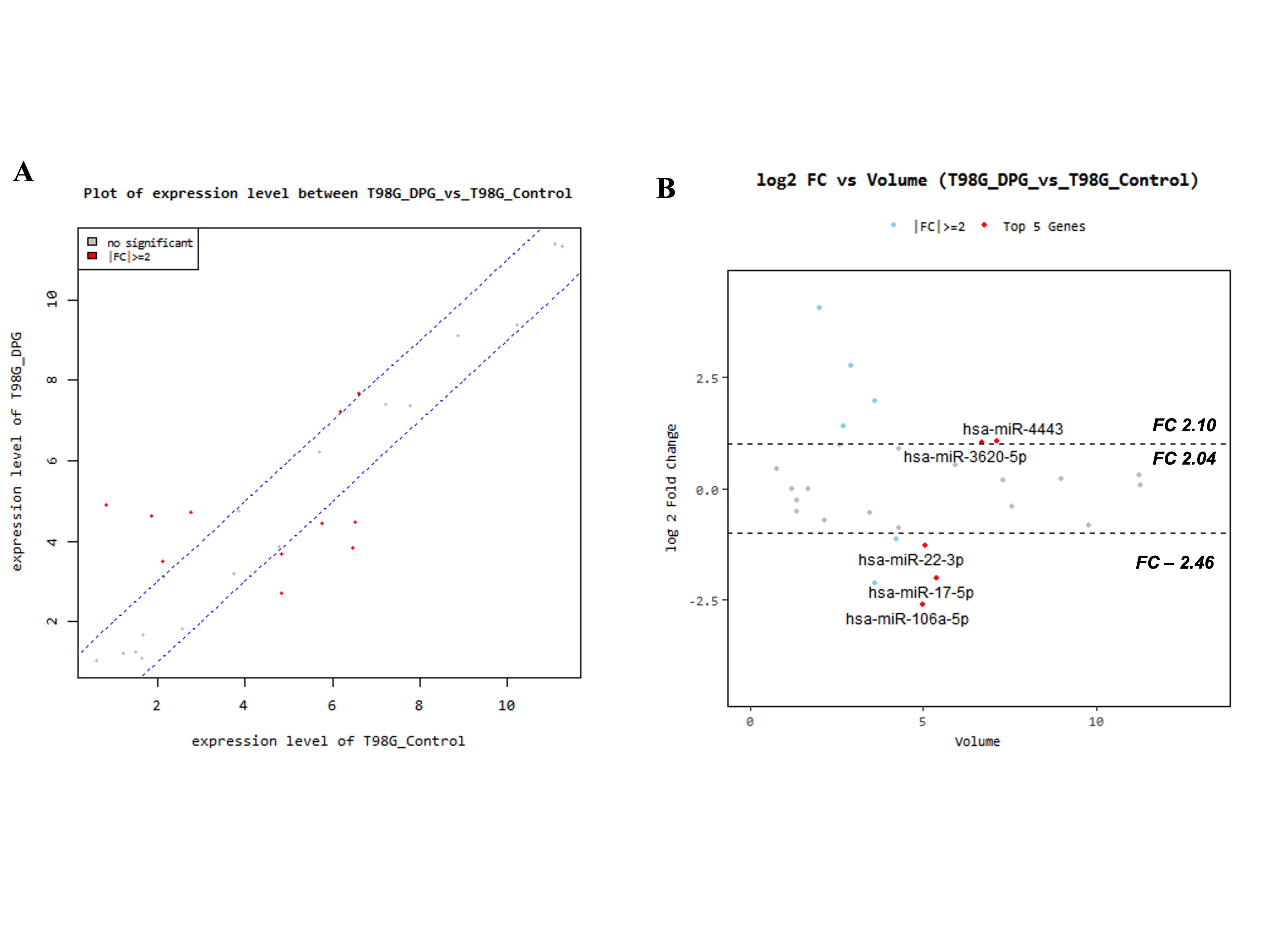

Supplement: Supplementary Figure 2 — Identification of microRNAs (miRNAs) with differential expression (DE-miRNAs) in glioblastoma T98G cell line. (A) Scatter plot of 11 DE-miRNAs identified after comparing dipotassium glycyrrhizinate (DPG)-treated and control T98G cell line (among 91 predicted miRNAs as NF-κB regulator genes). The gray and red dots represent each predicted miRNA. The red dots represent those that presented |fold change, FC ≥ 2|, according to the analysis of the computer program Affymetrix GeneChip® Command Console™. The dots above and below the dashed line represent those up- and downregulated miRNAs after DPG treatment, respectively. (B) Volume graph of the most overexpressed miRNAs among the 11 DE-miRNAs in T98G cell line exposure to DPG. Each gray, blue, and red dot represents one DE-miRNA. The red dots represent those miRNAs that presented both |FC ≥ 2| and increased volume of mRNA detected and quantified by the Affymetrix GeneChip® Command Console™ software. The dots above and below the dashed line represent those up- and downregulated miRNAs after DPG treatment, respectively. [file Image_2.jpeg]
