# Supplementary material for: Anti-Migratory Effect of Dipotassium Glycyrrhizinate on Glioblastoma Cell Lines: Microarray Data for the Identification of Key MicroRNA Signatures
Source: Front Oncol. 2022 Aug 3;12:819599. doi: 10.3389/fonc.2022.819599 (PMC9382584; doi:10.3389/fonc.2022.819599)
Supplement: Supplementary Table 1 — List of the 91 selected microRNAs predicted as NF-κB modulators and their predicted target genes (above 99%, according to TargetScan database) belonging to the NF-κB signaling pathway. [file Table_1.docx]

**Supplementary Table 1.** List of 91 selected microRNAs (miRNAs) predicted as NF-κB modulators and their predicted target genes (above 99%, according to Targetscan database) belonging to the NF-κB signaling pathway.

|  | **MicroRNAs** | **Target genes** | **Function** |
| --- | --- | --- | --- |
| 1 | miR-3173-3p | *CASP4* | Apoptosis - Caspase |
| 2 | miR-4448 | *CASP4* | Apoptosis - Caspase |
| 3 | miR-4698 | *CASP4* | Apoptosis - Caspase |
| 4 | miR-6829-5p | *CASP4* | Apoptosis - Caspase |
| 5 | miR-6089 | *TRAF2* | Apoptosis - Factor associated with the TNF receptor |
| 6 | miR-548ao-3p | *TRAF2* | Apoptosis - Factor associated with the TNF receptor |
| 7 | miR-20a-3p | *TRAF2* | Apoptosis - Factor associated with the TNF receptor |
| 8 | miR-544a | *TRAF2* | Apoptosis - Factor associated with the TNF receptor |
| 9 | miR-4790-3p | *TRAF2* | Apoptosis - Factor associated with the TNF receptor |
| 10 | miR-186-5p | *XIAP* | Apoptosis - Inhibitor of apoptosis |
| 11 | miR-4258 | *BAX* | Apoptosis - Pro-apoptotic |
| 12 | miR-6087 | *BAX* | Apoptosis - Pro-apoptotic |
| 13 | miR-4690-3p | *BAX* | Apoptosis - Pro-apoptotic |
| 14 | miR-5685 | *BAX* | Apoptosis - Pro-apoptotic |
| 15 | miR-766-5p | *BAX* | Apoptosis - Pro-apoptotic |
| 16 | miR-548u | *BCL2* | Apoptosis - Survival factor |
| 17 | miR-7161-5p | *BCL2* | Apoptosis - Survival factor |
| 18 | miR-4293 | *BCL2* | Apoptosis - Survival factor |
| 19 | miR-4713-5p | *BCL2* | Apoptosis - Survival factor |
| 20 | miR-4439 | *ABCB1* | Cell surface - Drug resistance by P-glycoprotein |
| 21 | miR-3136-5p | *ABCB1* | Cell surface - Drug resistance by P-glycoprotein |
| 22 | miR-466 | *ABCB1* | Cell surface - Drug resistance by P-glycoprotein |
| 23 | miR-626 | *ABCB1* | Cell surface - Drug resistance by P-glycoprotein |
| 24 | miR-183-3p | *ABCB4* | Cell surface - Drug resistance by P-glycoprotein |
| 25 | miR-4650-3p | *ABCB4* | Cell surface - Drug resistance by P-glycoprotein |
| 26 | miR-522-3p | *ABCB4* | Cell surface - Drug resistance by P-glycoprotein |
| 27 | miR-212-5p | *EGFR* | Cell surface - EGF receptor |
| 28 | miR-211-3p | *EGFR* | Cell surface - EGF receptor |
| 29 | miR-383-3p | *EGFR* | Cell surface - EGF receptor |
| 30 | miR-1238-3p | *TNC* | Cell adhesion - Control of cell growth, migration and adhesion by the ECM protein |
| 31 | miR-1587 | *TNC* | Cell adhesion - Control of cell growth, migration and adhesion by the ECM protein |
| 32 | miR-218-5p | *TNC* | Cell adhesion - Control of cell growth, migration and adhesion by the ECM protein |
| 33 | miR-3620-5p | *TNC* | Cell adhesion - Control of cell growth, migration and adhesion by the ECM protein |
| 34 | miR-4443 | *CD209* | Cell adhesion - Dendritic cell surface by leptin C |
| 35 | miR-4306 | *CD209* | Cell adhesion - Dendritic cell surface by leptin C |
| 36 | miR-185-5p | *CD209* | Cell adhesion - Dendritic cell surface by leptin C |
| 37 | miR-186-5p | *CD209* | Cell adhesion - Dendritic cell surface by leptin C |
| 38 | miR-3612 | *CD209* | Cell adhesion - Dendritic cell surface by leptin C |
| 39 | miR-1292-5p | *PTGS2* | Stress response - Production of cyclooxygenases and prostaglandins |
| 40 | miR-5698 | *MAP4K1* | Stress response - Activator of the stress-induced protein kinase pathway |
| 41 | miR-7111-5p | *MAP4K1* | Stress response - Activator of the stress-induced protein kinase pathway |
| 42 | miR-6870-5p | *MAP4K1* | Stress response - Activator of the stress-induced protein kinase pathway |
| 43 | miR-4723-5p | *MAP4K1* | Stress response - Activator of the stress-induced protein kinase pathway |
| 44 | miR-5004-5p | *MAP4K1* | Stress response - Activator of the stress-induced protein kinase pathway |
| 45 | miR-149-3p | *BCL3* | Transcription - Coactivator for NF-κB p50 and p52 |
| 46 | miR-27b-3p | *BCL3* | Transcription - Coactivator for NF-κB p50 and p52 |
| 47 | miR-4281 | *BCL3* | Transcription - Coactivator for NF-κB p50 and p52 |
| 48 | miR-4292 | *BCL3* | Transcription - Coactivator for NF-κB p50 and p52 |
| 49 | miR-4728-5p | *BCL3* | Transcription - Coactivator for NF-κB p50 and p52 |
| 50 | miR-6715b-5p | *E2F3* | Transcription - Cell cycle regulator |
| 51 | let-7a-2-3p | *GATA3* | Transcription - Cell differentiation factor |
| 52 | let-7g-3p | *GATA3* | Transcription - Cell differentiation factor |
| 53 | miR-1295b-5p | *GATA3* | Transcription - Cell differentiation factor |
| 54 | miR-1272 | *GATA3* | Transcription - Cell differentiation factor |
| 55 | miR-300 | *NFKBIA* | Transcription - Rel/NF-κB inhibitor |
| 56 | miR-1199-5p | *NFKBIA* | Transcription - Rel/NF-κB inhibitor |
| 57 | miR-143-5p | *NFKBIA* | Transcription - Rel/NF-κB inhibitor |
| 58 | miR-802 | *FOS* | Transcription - Proto-oncogene |
| 59 | miR-6728-5p | *FOS* | Transcription - Proto-oncogene |
| 60 | miR-106a-5p | *MMP3* | Enzymes - Related to metastasis |
| 61 | miR-106b-5p | *MMP3* | Enzymes - Related to metastasis |
| 62 | miR-17-5p | *MMP3* | Enzymes - Related to metastasis |
| 63 | miR-20a-5p | *MMP3* | Enzymes - Related to metastasis |
| 64 | miR-3123 | *MMP9* | Enzymes - Related to metastasis |
| 65 | miR-3713 | *MMP9* | Enzymes - Related to metastasis |
| 66 | miR-4691-5p | *MMP9* | Enzymes - Related to metastasis |
| 67 | miR-6734-3p | *MMP9* | Enzymes - Related to metastasis |
| 68 | miR-6792-3p | *MMP9* | Enzymes - Related to metastasis |
| 69 | miR-6764-5p | *TERT* | Enzymes - Telomerase catalytic subunit |
| 70 | miR-1915-3p | *TERT* | Enzymes - Telomerase catalytic subunit |
| 71 | miR-147a | *GADD45B* | Miscellaneous - DNA and cell cycle repairs |
| 72 | miR-3148 | *S100A4* | Miscellaneous - Tumor suppressor |
| 73 | miR-1273a | *S100A4* | Miscellaneous - Tumor suppressor |
| 74 | miR-1323 | *S100A4* | Miscellaneous - Tumor suppressor |
| 75 | miR-6745 | *S100A4* | Miscellaneous - Tumor suppressor |
| 76 | miR-1226-5p | *S100A10* | Miscellaneous - Related to annexin II |
| 77 | miR-1252-3p | *S100A10* | Miscellaneous - Related to annexin II |
| 78 | miR-1302 | *S100A10* | Miscellaneous - Related to annexin II |
| 79 | miR-3122 | *S100A10* | Miscellaneous - Related to annexin II |
| 80 | miR-4441 | *S100A10* | Miscellaneous - Related to annexin II |
| 81 | miR-22-3p** | *PTEN* | Miscellaneous - Tumor suppressor |
| 82 | miR-153-3p | *PTEN* | Miscellaneous - Tumor suppressor |
| 83 | miR-1179 | *VEGFC* | Growth - Vascular and endothelial growth factor |
| 84 | miR-1279 | *VEGFC* | Growth - Vascular and endothelial growth factor |
| 85 | miR-128-3p | *VEGFC* | Growth - Vascular and endothelial growth factor |
| 86 | miR-200a-5p | *VEGFC* | Growth - Vascular and endothelial growth factor |
| 87 | miR-10a-5p | *BDNF* | Growth - Brain-derived neurotrophic factor |
| 88 | miR-10b-5p* | *BDNF* | Growth - Brain-derived neurotrophic factor |
| 89 | miR-15a-5p | *BDNF* | Growth - Brain-derived neurotrophic factor |
| 90 | miR-16-5p | *BDNF* | Growth - Brain-derived neurotrophic factor |
| 91 | miR-195-5p | *BDNF* | Growth - Brain-derived neurotrophic factor |
